# Prediction of infectious diseases using sentiment analysis on social media data

**DOI:** 10.1371/journal.pone.0309842

**Published:** 2024-09-04

**Authors:** Youngchul Song, Byungun Yoon

**Affiliations:** Department of Industrial & Systems Engineering, Dongguk University, Jung-gu, Seoul, South Korea; American University of Beirut, LEBANON

## Abstract

As the influence and risk of infectious diseases increase, efforts are being made to predict the number of confirmed infectious disease patients, but research involving the qualitative opinions of social media users is scarce. However, social data can change the psychology and behaviors of crowds through information dissemination, which can affect the spread of infectious diseases. Existing studies have used the number of confirmed cases and spatial data to predict the number of confirmed cases of infectious diseases. However, studies using opinions from social data that affect changes in human behavior in relation to the spread of infectious diseases are inadequate. Therefore, herein, we propose a new approach for sentiment analysis of social data by using opinion mining and to predict the number of confirmed cases of infectious diseases by using machine learning techniques. To build a sentiment dictionary specialized for predicting infectious diseases, we used Word2Vec to expand the existing sentiment dictionary and calculate the daily sentiment polarity by dividing it into positive and negative polarities from collected social data. Thereafter, we developed an algorithm to predict the number of confirmed infectious patients by using both positive and negative polarities with DNN, LSTM and GRU. The method proposed herein showed that the prediction results of the number of confirmed cases obtained using opinion mining were 1.12% and 3% better than those obtained without using opinion mining in LSTM and GRU model, and it is expected that social data will be used from a qualitative perspective for predicting the number of confirmed cases of infectious diseases.

## Introduction

Infectious diseases are diseases that can spread from person to person and have continued to occur throughout human history. Since the first epidemic was recorded around 430 B.C., many infectious diseases have had huge impacts on mankind, such as the Black Death, smallpox, Spanish flu, and cholera. The Black Death killed approximately a third of Europe’s population, and smallpox has killed more than a billion people thus far. These disease epidemics have had major impacts on the overall economic conditions of the countries in which they occurred. COVID-19, which started in December 2019, has influenced many countries and has changed the lives of modern humankind. The World Health Organization (WHO) declared COVID-19 a pandemic, which is the highest risk level for infectious diseases, in March 2020. The declaration served as a starting point for the establishment of quarantine systems in each country in recognition of the severity of the pandemic. As human and property damage due to the COVID-19 pandemic increase [1], the pandemic can be classified as a social disaster that has caused large-scale damage at the national level. To date, the need to present health strategies for predicting infectious diseases and minimizing damage has emerged in the world, such as the implementation of distance-by-step and COVID-19 support policies.

With the increasing risk and impact of infectious diseases, researchers are uncovering the necessary data and methods to accurately forecast the number of confirmed cases. From a data perspective, most studies have employed daily confirmed case data to make predictions using regression or machine learning (ML) techniques [2–4]. In addition, some studies have been carried out to forecast the number of confirmed cases by identifying additional elements that influence the transmission of infectious illnesses, such as spatial data [5, 6]. However, there is a notable deficiency in integrating the subjective parts of social data, such as sentiment analysis, into models used for predicting infectious diseases. Thus, our study anticipates that including social data with these parameters will yield advantages.

This study begins with the assumption that the spread of infectious diseases is related to the sentiment polarity of social media. If a lot of negative sentiments are posted on social media, people will act more carefully, reducing the spread of the epidemic, and if the word "it’s okay" comes out a lot, people will be able to act casually and speed up the spread of the epidemic. When information pertaining to the risk of the coronavirus is spread through social networks, negative events can be transmitted through repeated exposure, resulting in acute stress [7]. The stress of this infectious disease causes people to change their behaviors to cope with it [8]. Since the start of COVID-19, people using social media data have been used to understand public psychological responses related to infectious diseases. In a survey, 93.3% of respondents stated that they avoid going to public places, 89.6% of the respondents reduced holiday-related activities, and more than 70% of the respondents stated said they take precautions to avoid infection [9]. Changes in people’s behaviors and the implementation of preventive measures in infected areas can affect the population density and quarantine, thereby curbing the spread of infectious diseases [10–12]. Therefore, it is considered meaningful to predict the number of confirmed infectious disease cases by analyzing people’s opinions pertaining to infectious diseases on social networks. This study aims to predict the number of confirmed cases of infectious diseases by using anonymized social media data containing collective public opinions on infectious diseases.

Considering this perspective, search volumes were used to predict the number of confirmed cases [13]. Sentiment analysis was conducted to explore the qualitative aspect of social data, and in [14], the number of future vaccinations was predicted on the basis of an setiment analysis of tweet data. To predict the number of confirmed infectious disease patients, daily numbers of confirmed cases and quantitative approaches to social and public data are being used. However, the above-referenced studies reflecting the qualitative characteristics of social data, which affect people’s psychology in terms of the number of confirmed infectious disease patients, are insufficient. Therefore, this study analyzes the qualitative characteristics of social data by means of opinion mining to check whether there exists a relationship between people’s sentiment states and prediction of the number of confirmed cases.

The motivation for this study lies in the observation that the social networking behavior of individuals can have an impact on the transmission of infectious diseases. Therefore, it is important to take this factor into account when forecasting the number of confirmed cases. This study utilizes data from social network services (SNS) to examine how the public responds to information about infectious diseases. It uses sentiment analysis, a method within the field of opinion mining, to analyze the sentiment expressed in these answers. The sentiment data that is retrieved is subsequently employed to forecast the quantity of confirmed cases of infectious diseases by utilizing machine learning models, with the objective of evaluating the accuracy of the predictions. The key findings of this study indicate that incorporating social media sentiment data into infectious disease prediction models results in better predictive performance compared to models that do not consider such data. This underscores the potential significance of social media data in improving the accuracy of infectious disease predictions. The study is structured as follows. Background explains the background theory of the contents covered in this study. Research Framework explains the research framework. The methods used herein are described in Results, and the results obtained using these methods are presented in Implications & Discussion. Finally, Conclusion presents the limitations and future directions of this research.

## Background

In this section, we review the extant literature on epidemic prediction, latest opinion mining processes, and ML models used for time-series prediction. First, we review how studies on infectious disease prediction have been conducted thus far, ML techniques used herein to predict the number of confirmed cases, and methods for opinion mining of social data.

### Predicting infectious diseases

To predict infectious diseases, Kemack and McKendrick proposed an infectious disease spread model by devising an SIR (Susceptible, Infectious, Recovered) model that considers uninfected, infected, and recovered people [15]. Assuming that all populations have the above population configuration, a series of differential equations were used to indicate the state of the overall population in terms of the number of infections. In this model, the formula was completed using the infection rate and recovery rate for each infectious disease, and studies on infectious diseases are still being conducted by using the SIR model and the modified SEIR (Susceptible, Exposed, Infectious, Recovered) model [16–18].

Moreover, in recent studies, with the advancement of artificial intelligence (AI), the number of confirmed infectious disease patients has been predicted using the ML and deep learning (DL) approaches, which are unlike the conventional model. The AI-based approaches consider diverse variables that affect infection, rather than merely considering the infection rate and recovery rate, which represent the unique characteristics of existing infectious diseases. This improves the prediction ability in dynamic situations. The number of confirmed cases in the early stages of COVID-19 was predicted using the ARIMA and TP-SMM-AR self-regression time-series models, respectively [19]. The Holt’s time series model was also used for forecasting confirmed cases, relying solely on global confirmed case data to predict future cases [4]. The ARIMA, Holt, Splines, and TBATS models were also used to predict confirmed cases, deaths, and cured cases of And USA and Italy [20]. In another study, simulations were conducted to create confirmed scenarios, and the impact and transmission order of spread were studied [5]. In studies using ML and DL, DNN, LSTM and gated recurrent unit (GRU) were used to predict the number of confirmed infectious disease patients [2, 6, 18, 21–24]. In addition, several ML techniques (K-nearest neighbor (KNN), support vector machine (SVM), and random forest (RF)) have been used to predict the number of people vaccinated [14]. The study exploited past pandemic case data to create a nonlinear autoregressive neural network time series model for forecasting confirmed cases. The studies primarily focused on making time series forecasts using solely confirmed case data, but also using other forms of data such as spatial data. While several studies have made predictions about the number of confirmed cases based on social data, they mostly relied on quantitative indicators obtained from social networks [13]. The models and data used in the previous studies are shown in Table 1. Some of these studies argue that social information can be analyzed for predicting confirmed infectious disease patients.

**Table 1:** Models and data used to predict the number of confirmed cases.

Model	Data	Reference
SIR	Daily confirmed cases	[16–18]
ARIMA	Daily confirmed cases	[19, 20]
Holt	Daily confirmed cases	[4, 20]
Simulation	Spatial data	[5]
ML&DL	Daily confirmed cases	[2, 18, 21–24]
	Mobility data	[6]
	Social data	[13, 14]

The best tools and data for predicting a dynamic epidemic such as COVID-19 are not specified. The data and tools that can be used to predict infectious diseases continue to be discovered to date. From the data perspective, a model that employs the results of opinion mining of social data can be tried.

### Opinion mining

Opinion mining is a big data analysis technique for analyzing and processing vast amounts of social text data. At the system level, it calculates the sentiment polarity of text sentences and is also called sentiment analysis. Many people read other people’s writings, and their behaviors are influenced by these writings, which can be analyzed through sentiment analysis [25, 26]. Sentiment analysis yielded significantly superior results on opinion-classification tasks than those of other text mining approaches [27]. Opinion mining can be used to identify people’s behavioral characteristics and expected phenomena through trend analysis and future prediction by using large numbers of opinions published on the Internet. The opinion mining of text data related to a specific topic facilitates the development of interesting approaches to the topic. An example is Obama’s successful 2012 election campaign, in which opinion mining was used, and analyses of buyers and users’ reviews by using opinion mining to gain insights in many customer analysis studies [28–30].

Usually, the process of opinion mining is as follows. First, the study targets are identified, and data with characteristics that the targets write or represent the target is collected and preprocessed. Thereafter, attributes such as opinions and attitudes, degrees of positivity/negativity, and satisfaction are used to select the characteristics to be extracted from the data. In the sentiment analysis conducted herein, positive/negative values are extracted, and to extract polarities, sentiment dictionaries and rule-based polarities are typically derived. Sentiment dictionary can analyze text data by using the words, rules, and polarities predefined in the sentiment dictionary to calculate positive/negative values depending on keyword appearance or rules [29–31]. Recently, a method of sentiment classification using ML and DL was studied [31].

Studies on the sentiment dictionaries used in sentiment analysis are being conducted. Because sentiment dictionaries use predefined values, it is important to build a sentiment dictionary that tailored to the corpus being analyzed. In previous studies, sentiment dictionaries were expanded successfully by using Word2Vec. Word2Vec is a word embedding technique that was introduced in 2013, and it uses a continuous bag of words (CBOW) learning method that predicts one blank by using multiple inputs and a skip-gram learning method that predicts surrounding blanks by using one input. The words learned in this manner have their respective vector values. In previous studies, the existing sentiment dictionaries were expanded using the cosine similarity of the Word2Vec results, and word dictionaries that were better optimized for the dataset to be analyzed were established [32–34]. In this study, sentiment analysis of social data is conducted by producing an extended sentiment dictionary by using Word2Vec in line with the changing characteristics of the existing sentiment dictionaries and social data.

### Machine learning

ML is being used in many predictive studies. ML is mainly divided into guidance, semi-supervised, and unsupervised learning depending on the learning method. Although ML is a black box model, meaning that how the model arrives at its results is not known, it is generally used in many fields such as recognition, classification, and prediction. Moreover, many predictive studies are underway to demonstrate strengths in the field of time-series prediction, and RNN techniques specialized for time-series analysis by remembering existing data are available. In addition, LSTM and GRU techniques have been derived from RNN. These models continue to be used for predicting infectious diseases. The present study aims to predict the number of confirmed infectious disease patients by using a deep neural network (DNN), a basic machine learning technique, in conjunction with LSTM and GRU specialized for time-series analysis.

**Fig 1:**
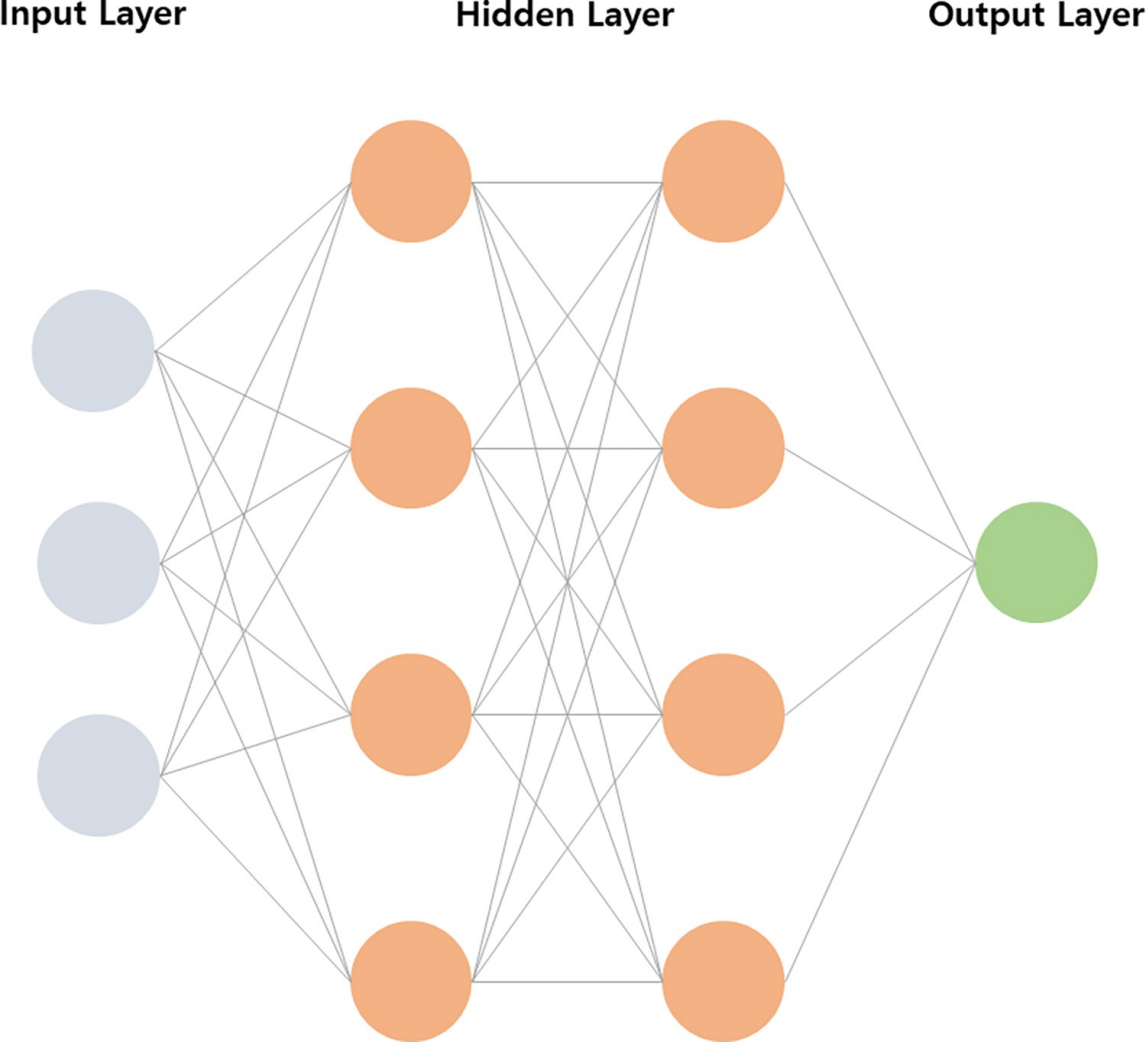
Structure of DNN.

A DNN is an artificial neural network that calculates outputs by multiplying weights across multiple hidden layers [35]. The DNN structure, illustrated in Fig 1, consists of an input layer, a hidden layer, and an output layer. These layers are connected to each other, and values are transformed and moved by using weights and activation functions. Each weight is modified by learning, and the network is created using the modified weights. DNNs are mainly used in supervised learning to solve classification and regression problems. When the predetermined learning process is completed, the result value of the new input value is derived using the final calculated weight. This DNN structure is also used for various tasks by connecting it to other ML techniques.

**Fig 2:**
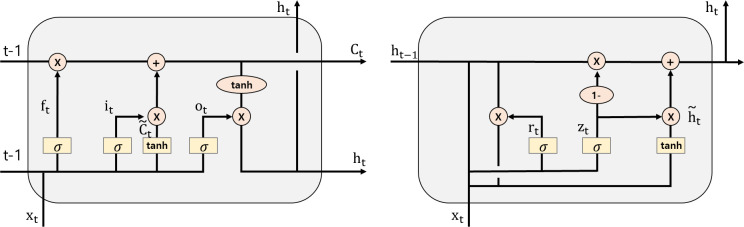
Structures of LSTM (left) and GRU (right).

LSTM is a circular neural network technique that was developed to overcome the limitations of RNN, which exhibits reduced learning ability owing to weak influence of past information [36]. The structure of LSTM is depicted in Fig 2, and LSTM learns by controlling the memory or by forgetting past information. In the figure, the flow of Ct refers to the cell state of the previous data; new information and previous ht are used to decide whether to preserve or discard information; input gate is added and multiplied using the sigmoid and tanh functions; and, finally, cell state is updated. In the output gate, ht is calculated using the sigmoid and tanh functions, which represents the short-term memory status and is identical to the value calculated in the corresponding cell and flowing out to the output. In conclusion, the result value is learned and derived using long-term memory, short-term memory, and new input information. LSTM with these characteristics is widely used for time-series analysis, and specifically, it is useful for time-series analysis involving volatility. The LSTM model has also been used from a time-series perspective in extant studies on predicting confirmed infectious disease cases [2, 6, 21].

The GRU model evolved from LSTM, and it simplifies LSTM to reduce learning time, thus resulting in similar performance but faster data learning [37, 38]. Unlike LSTM, GRU has a reset gate and an update gate, where the reset gate calculates the degree of reflection of the previous state (ht), and its role is similar to that of the forget gate. Meanwhile, the update gate determines the rate at which to reflect the previous state (ht) and the current input state (Fig 2). As with LSTM, the GRU model, too, has been used extensively for time-series analysis in recent years, and it has been used in studies on predicting the number of confirmed cases of infectious diseases [22, 24].

## Research framework

### Overall framework

**Fig 3:**
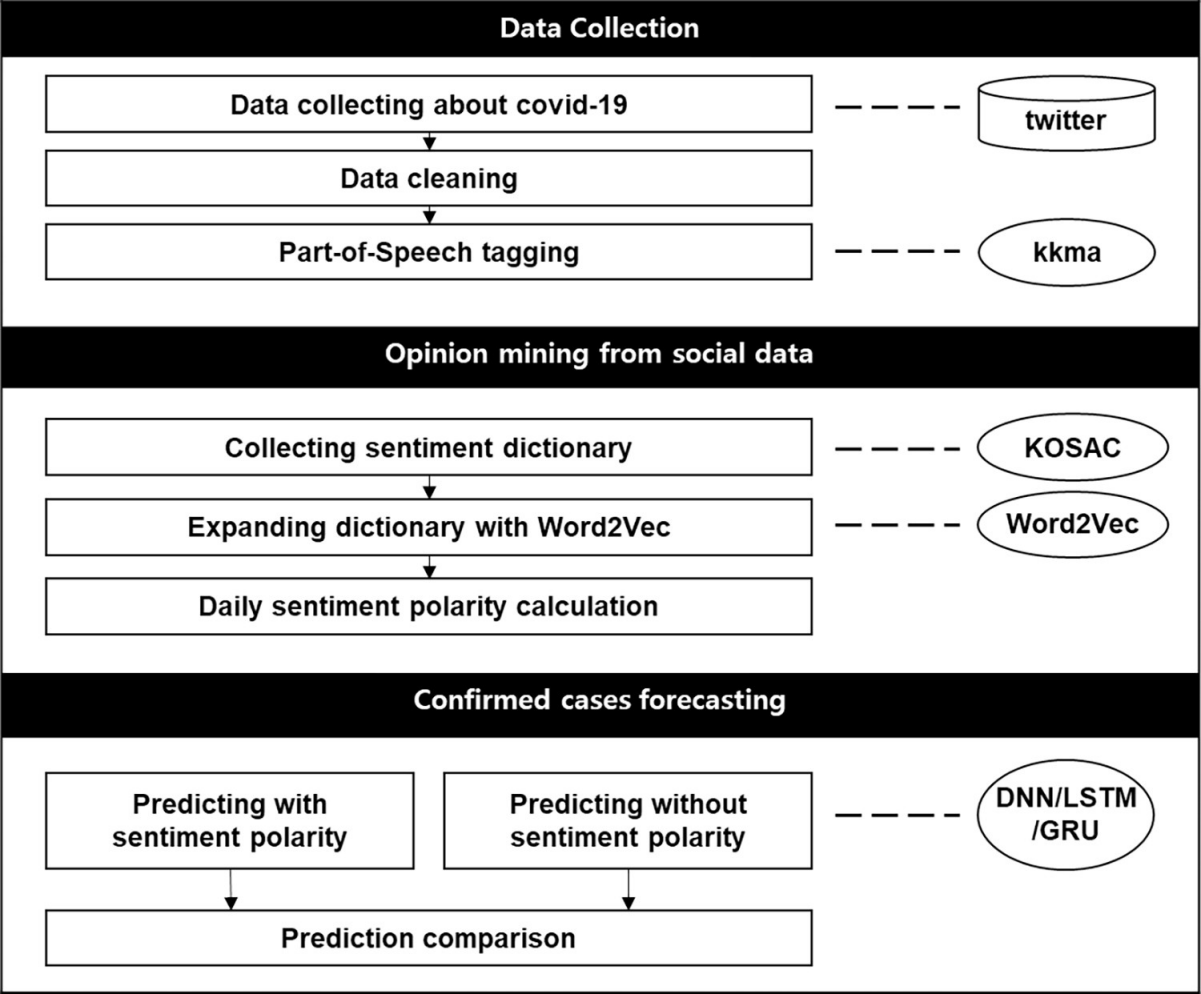
Overall framework.

In this study, data were obtained from Twitter, a social networking service (SNS) where one can freely write their thoughts, Pre-processing and part-of-speech (POS) tagging of these data were performed, and the positive/negative polarities of each tweet were derived daily using a sentiment dictionary. The number of confirmed cases was predicted through ML shown in Fig 3.

### Data collection and preprocessing

Among various SNS data, the tweet data of Twitter (https://twitter.com/) can be accessed by everyone. Moreover, people can freely express their thoughts on Twitter, and the amount of data on Twitter is adequate for analysis. Owing to these characteristics, this study preemptively found Twitter data to be suitable for use in this study. Tweet data containing keywords related to COVID-19 were extracted from Twitter. Tweet data of 30 months after the first confirmed case of COVID-19 were collected using Python by the collection and analysis method complying with the terms and conditions for the source of the data. The number of COVID-19 confirmed patients used in the study is collected at the Seoul Open Data Plaza (https://data.seoul.go.kr/). Duplicate data were deleted from the collected social data, and news data and promotional posts that did not contain user opinions were excluded. Thereafter, data in Korean only were created through preprocessing, and POS tagging was performed using Kkma.

### Opinion mining on social data

This study assumes that the information from social data can influence the spread of infectious diseases and that utilizing this data can lead to more accurate predictions of the number of confirmed cases. Therefore, the proposed methodology employs sentiment analysis of opinion mining to extract meaningful information from the social data. The opinion mining method used herein calculates the polarity of a sentence in terms of the average of polarities from the word perspective to determine the polarity of each text data. To start this process, it is necessary to define a sentiment dictionary to set the polarity of each word. Although a Korean-language sentiment dictionary is available, it has been expanded to match the characteristics of the SNS data collected using the Korean Sentiment Analysis Corpus (KOSAC) Korean sentiment dictionary [39], which, according to previous studies [27, 40], provides better results if a sentiment dictionary is written considering the characteristics of the each document.

In previous studies, the cosine similarity of Word2Vec was used to successfully expand the sentiment dictionary [32–34]. Therefore, in this study, the expansion of the sentiment dictionary using Word2Vec is confirmed to be necessary for better sentiment analysis. Polarities are determined based on the cosine similarity of words corresponding to positive/negative words by using the Word2Vec method. In case of the existing KOSAC Korean sentiment dictionary, each word has a label value for positive/negative as +1 for positive, -1 for negative, and 0 for neutral.

**Fig 4:**
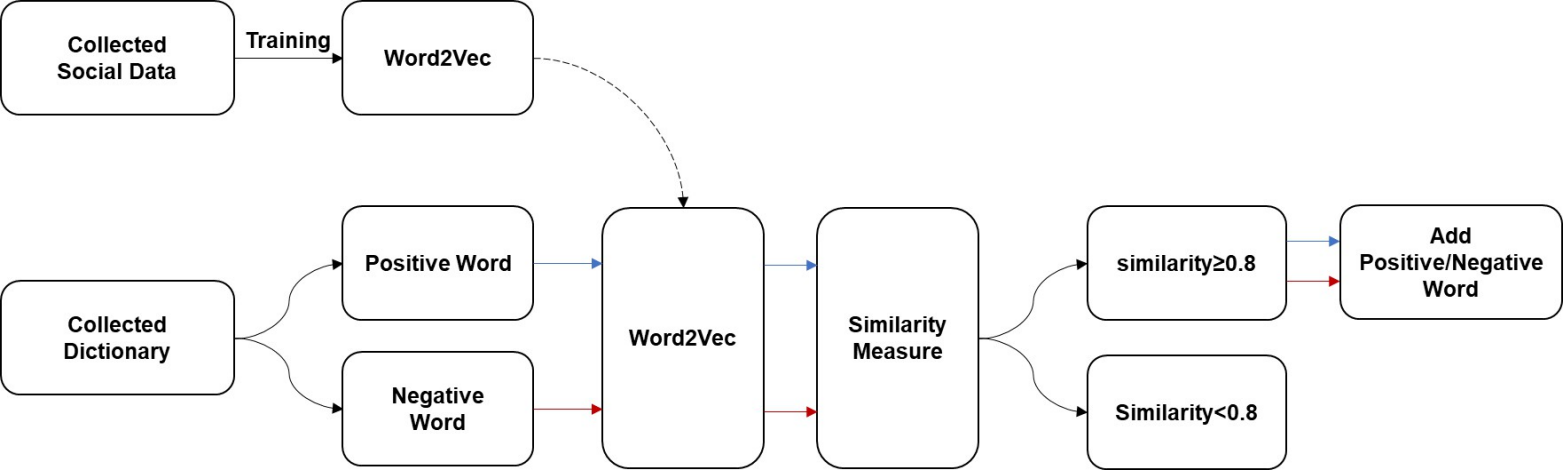
Diagram for expanding sentiment dictionary.

The Word2Vec model learned the collected 1.08 million text data. Between the CBOW and Skip-Bow learning models, we used the Skip-Bow model, which learns more data. This model was trained by setting the minimum number of appearances to 100, which was 0.01% of the amount of text data collected. By using the produced sentiment dictionary, positive/negative words and words with high cosine similarity were extracted by inputting words of sentiment dictionary into the Word2Vec model. Cosine similarity is calculated as shown in Eq1. Studies have demonstrated that a sentiment dictionary can be established successfully when the similarity is 0.5 or higher [34], and in this study, this study expanded the sentiment dictionary by considering a word an equivalent word with the same positive/negative label when the similarity of the word was 0.8 or higher to ensure high reliability (Fig 4). If a particular word originated from both positive/negative labels, the mean of cosine similarity was checked to provide a more similar positive/negative label.


$$Cosine\;Similarity(A,B)=\frac{A∙B}{\left\|A\|\|B\right\|}$$ (1)

The average polarity of each tweet was calculated by substituting the text data with adjectives, verbs, adverbs, nouns, and radix polarities in the produced sentiment dictionary (Table 2). Thereafter, the polarities of the daily text data were collected, and the daily polarity was calculated and used as the input to the model for predicting the number of confirmed patients. The formula for calculating the sentiment value of each tweet is given in Eq2. In Eq2, t represents each tweet, x represents the number of words in t that have sentiment polarities, and w represents the word in set x.


$$Sentiment\;Polarity(t)=\frac{1}{\left|x\right|}\sum_{w\in x}Polarity(w)$$ (2)

**Table 2:** Process of extracting text data polarity.

**Example of text data with positive polarity**	
…, ’world/NNG’, ’health/NNG’, ’organization/NNG’, ’ general/NNG’, ’is/JX’, ’accident/NNG’, ’Korea/NNG’, ’of/JKG’, ’Covid-19/NNG’, ’response/NNG’, ’method/NNG’, ’is/JX’, ’champion/NNG’, ’praise/NNG’, ’Korea/NNG’, ’of/JKG’, ’success/NNG’, ’Covid-19/NNG’, ’response/NNG’, ’is/JX’, ’government/NNG’, ’of/JKG’, ’democratic/NNG’, ’quarantine/NNG’, …	
**Average polarity**	**0.8**
**Example of text data with negative polarity**	
’headache/NNG’, ’hurts/VA’, ’and/ECE’, ’respiration/NNG’, ’also/JX’, ‘feel/VV’, ’heavy/XR’, ’electricity/NNG’, ’mat/NNG’, ’open/VV’, ’and/ECE’, ’lie/VV’, ’Covid-19/NNG’, ’headache/NNG’, ’Covid-19/NNG’, ’upset/VA’, ’stomach/NNG’, ’drama/NNG’, ’watch/VV’, …	
**Average polarity**	**-0.91**

### Predicting number of confirmed cases

**Fig 5:**
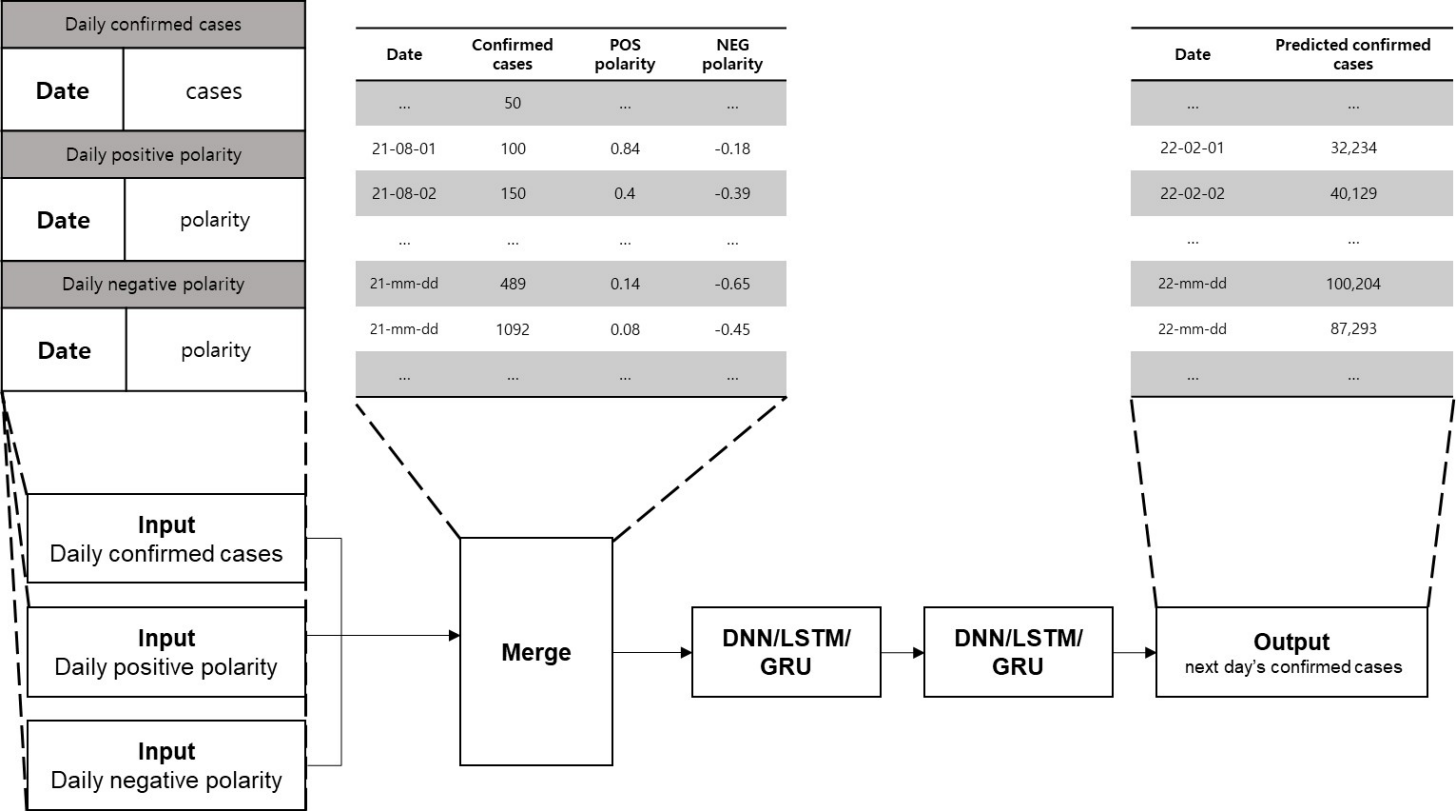
Layer configuration diagram.

**Fig 6:**
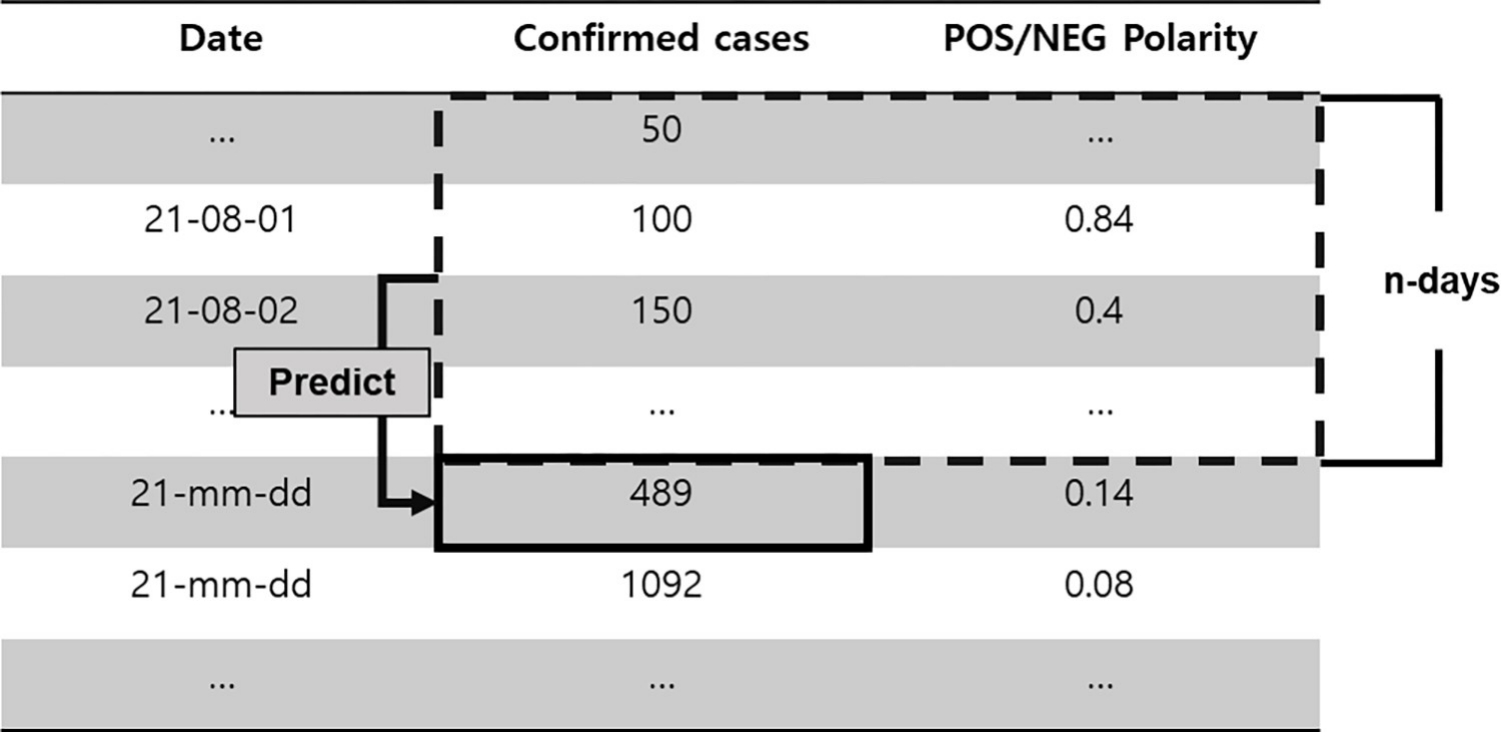
Machine learning prediction method.

Based on successful cases of predicting the number of confirmed cases using machine learning, this study also employs models from the machine learning family (DNN, LSTM, GRU) that have demonstrated high effectiveness [2, 6, 18, 21–24]. In this part, predictions with and without daily positive/negative polarities obtained from opinion mining are compared. First, predictions were generated using the DNN, LSTM, and GRU models by using only the number of confirmed patients per day, and predictions were generated under the same conditions by including the positive/negative polarities. To compare the prediction accuracy in this process, the Mean Absolute Percentage Error (MAPE), which calculates the ratio of the difference between the predicted value and the actual value according to the characteristics of the number of confirmed patients with a large range, was used. To predict the number of confirmed cases of infectious diseases, the DNN, LSTM, and GRU ML models consisting of two hidden layers, as shown in Fig 5, were applied to finally predict linear values. The data used for prediction were the daily positive/negative polarities extracted in opinion mining on social data part and the data on the number of confirmed patients in Korea. These data were divided in a 7:3 ratio into the learning dataset and verification dataset, and the prediction model was applied to these two datasets. An example of input data is depicted in the blue box in (Fig 6). After predicting the number of confirmed cases on the next day by using the daily number of confirmed cases and positive/negative polarities of n-days before the forecast date, the MAPE values of the actual and predicted values were calculated to measure the prediction accuracy.

Before executing the final prediction algorithm, the number of confirmed cases and the daily polarity calculated in opinion mining on social data part, were applied to the model as input values, and the optimal model and duration were confirmed by conducting several experiments. Subsequently, in this study, the predicted number of confirmed cases on the next day obtained by using only the data of the number of confirmed cases and the prediction results obtained using daily polarities are compared to confirm the prediction accuracy (Fig 5). The input data are used as daily polarities, and the number of confirmed cases of n-days before the forecast date and MAPE values are calculated by comparing the predicted and actual values of the next day to confirm the results.

## Results

### Data collection and preprocessing

Search terms were collected using a total of five words, including four Corona-related words (“Corona,” “COVID-19,” “COVID-19 confirmed and “COVID-19 Vaccine” based on Google Trends) and “epidemic.” Prior to collecting data for machine learning techniques, this study considered whether a small amount of data could be used. To measure the daily number of confirmed cases of infectious diseases, data from when the epidemic is active should be used, because there were numbers of units that did not fit perfectly in the category of big data. However, recent papers predicting the number of confirmed cases of infectious diseases using machine learning have also been confirmed using a small amount of data like Table 3. Therefore, although limited in this study, the prediction was conducted using 756 points of data. In addition, fields that require actual infectious disease prediction will also require rapid response, and the model proposed in this study reflects situations in which they are forced to use less data.

**Table 3:** Prior literatures’ data period examples.

Literature	Data period (point)
[2]	2020.01.20–2020.12.21(336 points)
[22]	2020.01.06–2022.06.06(882 points)
[23]	-2020.10.25(250 points)
[24]	2021.12.20–2022.11.14(329 points)

The data-collecting period spanned from February 24, 2020, to March 21, 2022. A total of 1,080,000 data points were obtained after undergoing preprocessing procedures to exclude duplicate or missing information, as well as advertisement messages from the social media site (Twitter). The collected data include both the date and the corresponding text generated. A total of 1,423 data points were gathered on a daily basis, with a standard deviation of 318.23. Furthermore, data regarding the number of confirmed COVID-19 cases in Korea within the aforementioned time frame was also gathered. POS tagging of these text data was performed using a Kkma POS tagger, and finally, the data were produced, as summarized in Table 4.

**Table 4:** Example of preprocessing results.

Date	Text
2022-03-30	epidemic/NNG era/NNG table/NNG unnecessarily/MAG …
2022-03-30	honestly/MAG reminder/NNG badly/MAG not/MAG like/VV …
2022-03-30	china/NNG in/NNB epidemic/NNG spread/NNG through/JKM …
2022-03-30	I/NP am/JX this/MDT big/VA, epidemic/NNG is/JX …
2022-03-30	continued/NNG 6/NR hour/NNG little/MAG short/VV …
2022-03-30	this/NNG epidemic/NNG through/JKM several/VA, days/NNG …

### Opinion mining on social data

To match the data collected in the KOSAC Korean sentiment dictionary and the social data, a sentiment dictionary was produced using the Word2Vec technique. Before Word2Vec was used, it learned the entire POS-tagged text data summarized in Table 1.The minimum number of appearances was 100, which accounted for 0.01% of the total sentence data, and the Skip-Bow model was used as the learning method. As the input data, words from the KOSAC sentiment dictionary were inserted, and words with a cosine similarity of 0.8 or higher, derived through Word2Vec, were added to the new sentiment dictionary because they were considered to have the same positive/negative sentiment polarities. To account for the morphemes of the words, an sentiment dictionary comprising nouns, verbs, adverbs, and adjectives was collated, and a total of 3,070 sentiment words and values were finally extracted (Table 5).

**Table 5:** Example of expanded sentiment dictionary.

Sentiment dictionary.			
Positive word (+1)		Negative word (-1)	
Comfortablity/NNG	deposit/NNG	Monololy/NNG	headache/NNG
antibacteria/NNG	achievement/NNG	illness/NNG	fever/NNG
happiness/NNG	affectionatly/XR	reproach/NNG	solitude/NNG
hopeness/NNG	release/VV	regression/NNG	sick/VV
calmness/NNG	recognize/VV	destruction/NNG	failure/VV
save/VV	confidently /MAG	rough/XR	tragic/NNG
full/XR	reliable/XR	disgusting/XR	inevitable/XR

**Fig 7:**
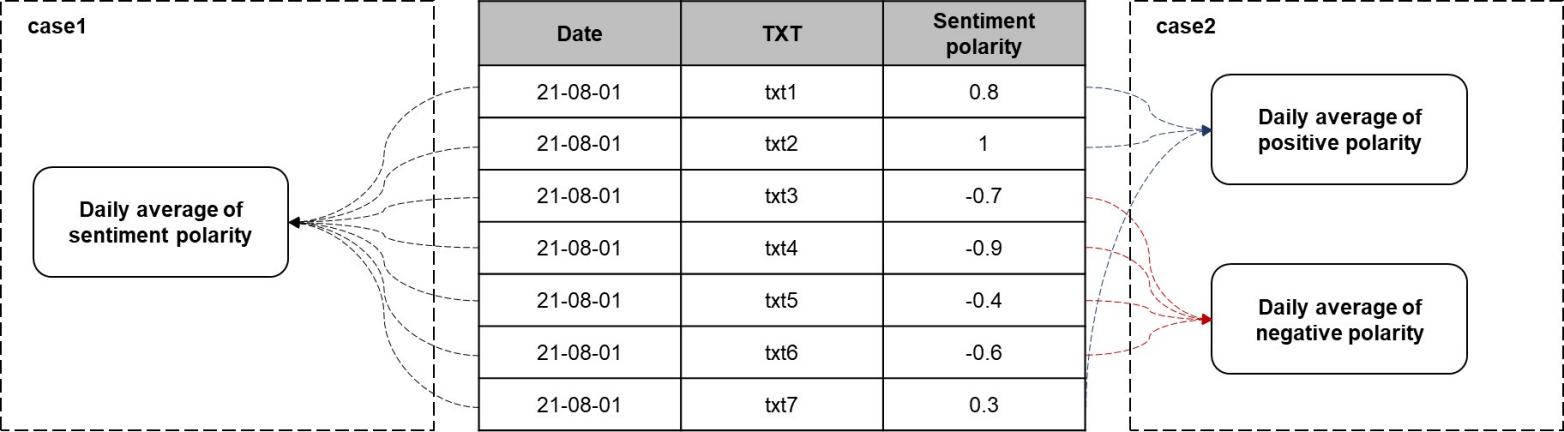
Method for calculating daily polarities.

The average of polarities was calculated for each text data collected using the produced sentiment dictionary. The decision was made considering the two methods used to calculate the daily polarity values from the text data polarity values. As illustrated in Fig 7, Case 1 has positive and negative sentiment polarities from -1 to 1 on each date, and Case 2 uses two input data that are calculated daily by separating texts with positive polarities from those with negative polarities.

- Case 1: Using the average of daily polarities
- Case 2: Using the mean of each positive and negative daily polarities

The final calculation method was the one that yielded the better prediction results in terms of the number of confirmed infectious disease patients. As a comparative index of the final prediction result, the MAPE values of the predicted and measured values were used, and the results are summarized in Table 6. In terms of minimum value, the MAPE values were 11.57% in Case 1 and 10.09% in Case 2. Therefore, as indicated by Case 2 in Fig 7, the method of calculating the polarity by dividing it into positive and negative was adopted. Table 7 summarizes the polarity of each text data, and Table 8 is a normalized table containing the average values obtained by dividing the daily polarity by positive and negative polarities. The daily polarity represents the degree of positive/negative COVID-19-related opinions of users in the text data obtained from SNS on the corresponding date, and it is finally input into the prediction model in the form of Table 8.

**Table 6:** Prediction accuracy results by case.

Case 1			Case 2		
DNN	LSTM	GRU	DNN	LSTM	GRU
**12.569%**	13.611%	14.268%	12.945%	11.999%	**10.093%**

*Each MAPE value is an arithmetic average of 30 learning and test runs.

**Table 7:** Example of extracted text polarity data.

Date	Text	Polarity
2020-12-11	’I/NNG’, ’TT/EMO’, ’also/MAG’, ’covid-19/NNG’, ’still/MAG’, ’be/VV’, ’not/MAG’, ’go/VV’, ’like/VA’, …	-1
2020-12-11	’curious/XR’, ’thing/NNB’, ’is/JKS’, ’exist/VV’, ’the/MDT’, ’USA/NNP’, ’in/JKM’, ’live/VV’, ’and/ECS’, ’come/VV’ …	-0.25
2020-12-11	’daughter/NNG’, ’also/JX’, ’do/VV’, ’speech/NNG’, ’is/JKS’, ’daily/MAG’, ’subway/NNG’, ’at/JKM’, ’closely/MAG’, ’kiss/NNG’, …	-0.80952
2020-12-11	’nice/VA’, ’meet/VV’, ’covid-19/NNG’, ’care/NNG’, ’do/XSV’, ’and/ECE’, ’always/MAG’, ‘happiness/NNG’, …	1
2020-12-11	’covid-19/NNG’, ’certainly/MAG’, ’we/NP’, ’hazard/NNG’, ’give/VV’, ’hope/VV’, ’but/ECE’, ’TT/EMO’, ’guarantee/NNG’, …	0.555556
2020-12-11	’LAN/NNG’, ’line/NNG’, ’meeting/NNG’, ’fun/NNG’, ’use/VV’, ’video/NNG’, ‘cam/NNG’, ’through/JKM’, ’wine/NNG’, ’seat/NNG’, …	0.6

**Table 8:** Example of a table of daily polarities.

Daily polarities		
Date	Positive polarity	Negative polarity
**2022-03-16**	0.45017	-0.720945
**2022-03-17**	0.477276	-0.654948
**2022-03-18**	0.379162	-0.623584
**2022-03-19**	0.442367	-0.781179
**2022-03-20**	0.487673	-0.942636
**2022-03-21**	0.586816	-0.99777
**2022-03-22**	0.297567	-0.327079
**2022-03-23**	0.124204	-0.345326
**2022-03-24**	0.695178	-0.530566

### Predicting the number of confirmed cases

In this section, the number of confirmed cases is predicted using DNN, LSTM, and GRU, which are the machine learning models proposed in the research framework. The input values of the model include the number of confirmed cases in Korea between February 24, 2020, and March 21, 2022, which is the period when the number of confirmed cases appeared steadily in Korea; number of confirmed cases; and positive/negative polarities derived through opinion mining. The data were divided in a ratio of 7:3 to obtain the training and verification datasets, and learning was performed. As for the activation function of DNN, the RELU function with the best results was applied after comparing the experimental results of the sigmoid, RELU, and softmax models; the epoch of each model was set to 500, and learning was performed. The results were confirmed using the Adam optimizer, which yielded the best experimental results among the candidate optimizers, namely Root Mean Square propagation(RMSP), Stochastic Gradient Descent(SGD), Adaptive Moment Estimation(Adam), and Nesterov Accelerated Gradient Adam(Nadam).

The prediction results were organized, as shown in Table 9, depending on whether the daily polarities were included and by considering the scope of data application. Depending on the presence or absence of polarities, the daily polarity data were divided into applied and notapplied. The prediction inclusion period was used to set the number of data matches required to generate predictions based on the prediction date. For example, if the prediction inclusion period was 14, the value of the prediction point was calculated using the data of 14 days, including the day before the prediction point. In this study, 7 days, the average incubation period expected by the Korea Centers for Disease Control and Prevention; 14 days, the longest officially announced incubation period; and 28 days, the period considering the impact of the previous incubation period due to the nature of the epidemic were used. The MAPE, MSE, RMSE, MAE results summarized in Table 9 were expressed as the average of 30 prediction results. The number of confirmed cases of infectious diseases has an exponential characteristic. Therefore, if the results are presented using only error figures such as MSE, RMSE, and MAE, the MAPE value that can be expressed as a ratio of errors is presented in this study because a model that performs prediction well may be judged to be better when the number of confirmed cases is relatively large.

**Table 9:** Model accuracy results.

**Variable**		**MAPE**			**MSE**		
**Polarity present**	**Prediction duration**	**DNN**	**LSTM**	**GRU**	**DNN**	**LSTM**	**GRU**
**Polarity**	7	**12.95%**	13.00%	12.86%	352,820,600	327,171,199	328,005,526
**excluded**	14	13.21%	12.93%	13.03%	366,212,960	319,480,205	315,897,221
	28	13.02%	13.18%	12.56%	349,218,400	328,637,013	339,790,403
**Polarity**	7	13.77%	20.43%	19.71%	338,338,340	664,817,539	609,436,773
**included**	14	14.49%	**12.00%**	**10.09%**	343,742,800	362,054,368	283,949,449
	28	14.41%	13.54%	18.12%	355,748,670	404,569,246	517,321,834
**Variable**		**RMSE**			**MAE**		
**Polarity present**	**Prediction duration**	**DNN**	**LSTM**	**GRU**	**DNN**	**LSTM**	**GRU**
**Polarity**	7	18,762	18,087	18,103	6,017	5,897	5,728
**excluded**	14	19,108	17,874	17,771	6,211	5,816	5,568
	28	18,677	18,125	18,415	6,129	6,026	5,879
**Polarity**	7	18,388	25,778	24,684	6,101	8,208	7,804
**included**	14	18,525	18,901	16,837	6,377	5,865	5,071
	28	18,837	19,928	22,712	6,445	6,655	7,605

* Each MAPE value is an arithmetic average of 100 learning and test runs.

**Fig 8:**
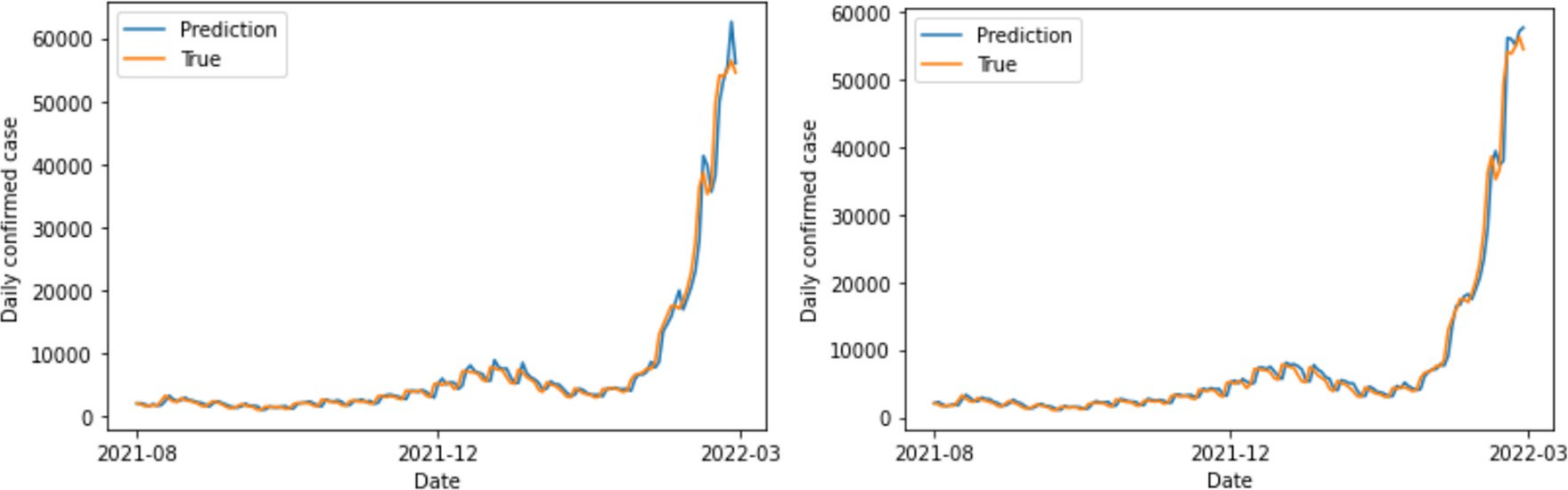
DNN results obtained using 14-day data with polarity excluded (left) and included (right).

**Fig 9:**
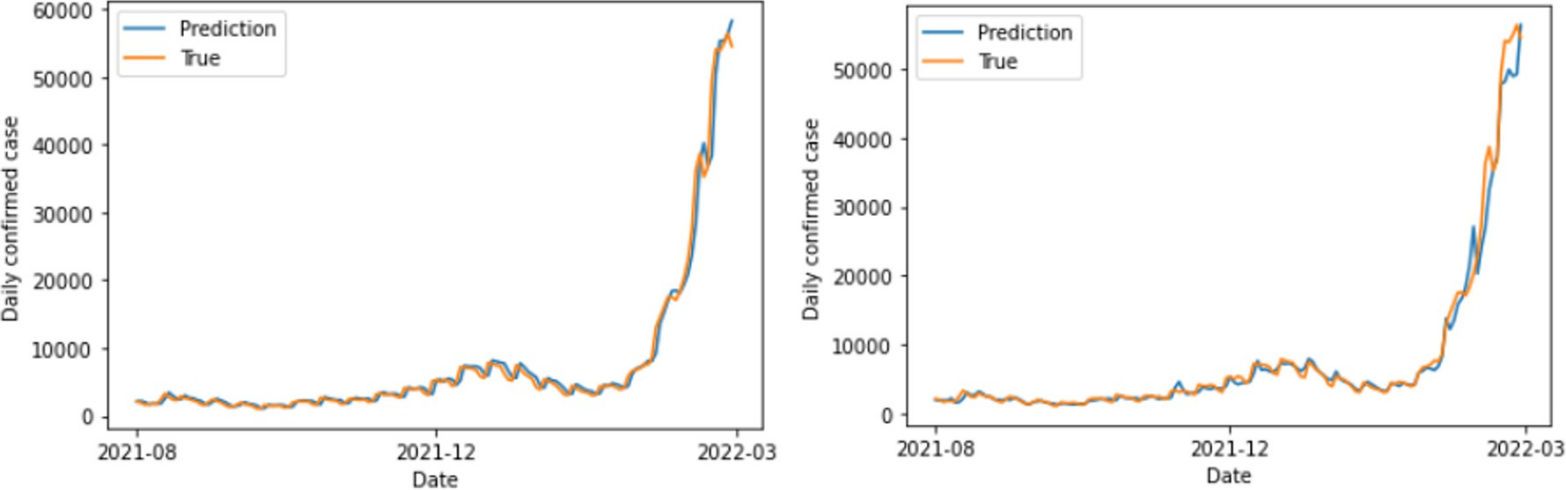
LSTM results obtained using 14-day data with polarity excluded (left) and included (right).

**Fig 10:**
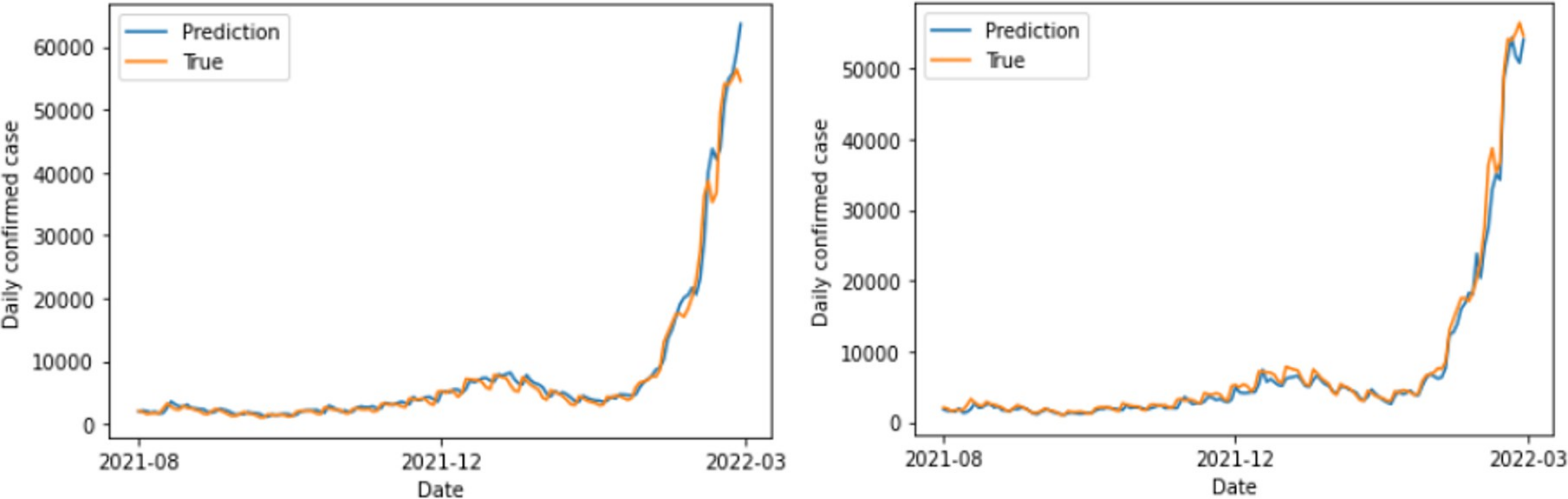
GRU results obtained using 14-day data with polarity excluded (left) and included (right).

The study found that the GRU model achieved the lowest error rate value of 10.093%, including polarities, for a 14-day period. This aligns with the expected incubation period for COVID-19 (1–14 days) announced by the Korea Centers for Disease Control and Prevention. Furthermore, for DNN, the data without polarities exhibited greater predictive power (Fig 8). Conversely, the RNN family models—LSTM and GRU—achieved satisfactory prediction outcomes when utilizing data that had polarities (Figs 9 and 10). A t-test was performed to compare the accuracy of 100 learning/test runs using LSTM and GRU models on 14-day data. The comparison was done using both data sets, with and without sentiment polarities. The t-tests resulted in p-values of 1.28e-09 for LSTM and 5.92e-153 for GRU. These values indicate that the results obtained from data that included polarities were statistically significantly superior than those obtained from data that excluded polarities. The analysis and evaluation of 100 learning/test runs highlight the strength and reliability of the findings.

In addition to the t-test, a binomial test was performed to verify the statistical significance of the win/loss information for each trial. This is crucial because the proposed strategy might "lose" more comparisons but still have a lower average, or alternatively, "win" more comparisons in both the 14 days and 28 days settings but have a lower average in the 28 days setting. For the LSTM results over a 14 days period, the model that included polarities won 82 out of 100 comparisons. This result allowed to reject the null hypothesis that the win probabilities of the two models are equal, with a p-value of 6.14e-11. In the 14 days GRU comparison, which demonstrated the best predictive performance, the model including polarities won all 100 comparisons. These results strongly support that the proposed feature is more significant when it comes to the actual model training. This analysis confirms the effectiveness of the proposed strategy and highlights the importance of incorporating polarities into the model for better predictive performance.

This study also compares its results with other research methods. This work selects the ARIMA model, which utilizes machine learning to make predictions based on time series data [19, 20]. Prior research has indicated that the ARIMA model outperforms the Holt, Splines, and TBATS models in predicting the number of confirmed cases on weekly intervals [20]. Hence, in order to assess performance, this study used the approach of forecasting the weekly count of confirmed cases and thereafter comparing the results. The comparison is made by displaying the MAPE values at weekly intervals starting from the initial prediction date [20]. The ARIMA model, which demonstrated superior accuracy in prior research, is being compared by the results obtained for situations with and without sentiment polarity. The model’s performance is adequate for forecasting the number of COVID-19 cases in Korea and was evaluated using the ARIMA (2,1,3) parameters suggested in [41]. Table 10 shows the MAPE values for these models during a six-week period starting from the prediction’s initial date. It also presents a comparison of their average values over the entire period. On average, the GRU model outperformed the ARIMA model in terms of MAPE performance, as indicated by the comparison results. In addition, while evaluating the average performance over the entire period, it was found that the GRU model outperformed the ARIMA model (Table10). This study examines the impact of incorporating sentiment polarity on the quality of results. The trials utilizing the ARIMA model also indicate that the results, which incorporate the sentiment polarities, show some improvement. Furthermore, with the exception of the data from Period1, the study consistently validated that the models incorporating GRU and sentiment polarity had superior performance on average. This comparison highlights the significance of taking sentiment polarity into account when making predictions. It demonstrates that the findings obtained by including sentiment polarity had reduced MAPE values, even when it is used in the method of previous studies.

**Table 10:** Comparison of prediction model results at 7-day intervals.

Model	Sentiment Polarity	Period1 Sep19-Sep25	Period2 Sep26-Oct2	Period3 Oct3- Oct9	Period4 Oct10- Oct16	Period5 Oct17- Oct23	Period6 Oct24- Oct30	Period1-6 Average	Entire Period Average
ARIMA	Excluded	15.33%	11.95%	14.35%	19.09%	13.04%	13.02%	14.46%	16.98%
	Included	15.29%	11.83%	13.86%	19.07%	13.24%	12.42%	14.29%	16.89%
GRU	Excluded	10.92%	13.69%	16.04%	15.32%	14.97%	11.84%	13.80%	13.03%
	Included	15.70%	9.08%	9.41%	9.06%	7.37%	6.59%	9.53%	10.09%

## Implications & discussion

The results of this study indicate whether the qualitative opinions in social data were considered when predicting the number of confirmed infectious disease patients. In addition, the prediction results obtained using various ML models (DNN, LSTM, GRU) are presented. Finally, the best predictive power was obtained when the GRU model was applied to the data that included polarities. Moreover, all RNN family models yielded statistically significantly better predictive results when using the data that included polarities. According to the LSTM and GRU prediction graphs in Figs 9 and 10 obtained using the data that included or excluded polarities, respectively, the predicted values are smooth when the polarities are excluded, but they have trailing graphs. Trailing graphs indicate low efficiency in real environments. Trailing graph responds late to the forecast flow because it is similar to the amount of data immediately preceding it. This can make it difficult to utilize the prediction results. By contrast, when the polarity is included, the graph is relative rough, but it seems to yield a predictive value that is appropriate for the timing. In addition to the MAPE set as the error value, the characteristics of the graph showed more remarkable results. In addition, the results were compared using the ARIMA model among previous research methods, and it was also confirmed that the model with GRU and sentimental polarity showed the best performance. Therefore, according to our study, better predictive are generated by considering the qualitative characteristics of social data in the prediction process. Additionally, in this study, a model was developed to reduce errors in the predicted and measured values of the number of confirmed cases, but it is expected that it will be developed as a more effective model if a model for rise and fall is presented for future purposes.

During the research process, two methods for calculating the daily polarity were proposed to predict the number of confirmed patients. The first method involved viewing all polarities as an average for each day, and the second method involved calculating the positive and negative polarities separately. As a result of the experiment, the average was obtained by dividing the positive and negative polarities, and when this method was applied to the prediction model, the prediction accuracy increased. The reason for the application of this method was that if multiple data were to be combined using the central limit theorem, the value would remain at a certain level, which would reduce the data dimension that could be expressed for each degree. Moreover, the results were superior when multiple data were included. In future studies on opinion mining and sentiment analysis, it will be possible to consider the method of using polarities by dividing positive and negative properties. In this study, when applying opinion mining to social data, only the method that considered the frequency of words in the existing sentiment dictionaries was used. In future research, this part will be supplemented to reflect advanced research on opinion mining methods. Recently, with the advancement of NLP in the opinion mining and sentiment analysis domains, many studies have been conducted. For example, studies that measure polarities of social data through the use of Transformers, including BERT, are actively underway, and if these tools can analyze polarities from various angles and reflect them, more useful and improved research results can be expected.

It was also meaningful to confirm the data period for predicting the number of confirmed cases in this study. The incubation period proposed by the Korea Centers for Disease Control and Prevention was considered to determine the period for including previous data as the input data before generating predictions using the ML model. The Korea Centers for Disease Control and Prevention announced that the average and maximum incubation periods were 7 days and 14 days, respectively. Therefore, this study was conducted for up to 28 days in consideration of the average incubation period of 7 days, longest incubation period of 14 days, and the 14 day period before the infected person was affected. According to the study results, the LSTM and GRU models yielded the best predictions when using 14day data that included polarities. The meaning of 14 days overlaps with the meaning of 2 times the average incubation period of 7 days suggested by the Korea Centers for Disease Control and Prevention and the maximum incubation period of 14 days. These results suggest that further analysis is necessary to determine the significance of the relationship between the incubation period announced by the Centers for Disease Control and Prevention and the use of social data to predict infectious diseases.

In the social data covered intensively in this study, new words or new expressions appear over time owing to the characteristics of language. In this study, this study proposed a method for including these expressions in sentiment analysis by developing an existing sentiment dictionary using Word2Vec. This method can automatically collect data that reflect the changing characteristics of SNS language without needing a qualitative process involving experts. In addition, it is possible to update the sentiment dictionary to reflect the newly emerging language trends and conduct sentiment analysis automatically. This feature ensures that the proposed model can be updated and applied at a certain point in time in the future. In order to utilize the results of this study, users can collect social data containing the degree of positivity to infectious diseases and use the extracted sentiment polarities of each content as a parameter for infectious disease prediction algorithms. In order to extract the sentiment polarity of each data, an sentiment dictionary must be established considering the characteristics of each language, and it is expected that analysis can be performed according to the characteristics of each country and epidemic spread. Predicting the number of confirmed cases of the pandemic will keep individuals alert, enable policymakers to pre-imagine health-related resources and personnel plan, and allow them to move toward a quick end to the pandemic, taking into account when planning a response to preventive measures to prevent it.

Notwithstanding these contributions, it should be noted that the findings being given are applicable only to particular places and circumstances. This study employed qualitative aspects of social data to forecast the number of confirmed instances of infectious illnesses. To ensure accurate utilization, it is important to account for the amount of people engaged in social data and the regional influence of such data. Furthermore, it is important to incorporate variations in language and grammar structures, disparities in social media usage and recognition patterns, as well as cultural norms and frequency of social media engagement across different nations, since these factors can significantly impact social media dynamics and user behavior. This article presents the findings of a research endeavor that involved the development and validation of an epidemic prediction model. The model was constructed by leveraging opinion mining outcomes derived from social data in Korea, a country characterized by dense population and extensive utilization of social network services. In the future, it will be necessary to construct models using opinion mining in various languages and nations.

## Conclusion

This study aimed to propose a methodology for predicting the number of confirmed cases of infectious diseases by using opinion mining, which allows for the inclusion of qualitative opinions from social data in epidemic prediction. To this end, about 1 million SNS Twitter data were collected, and the Word2Vec model was learned using the collected social data to expand the existing sentiment dictionary for sentiment analysis. After that, a model was developed to predict the number of confirmed COVID-19 patients by using the calculated sentiment polarities, and predictions were generated. As a result, when predicting using sentiment polarities, the predictive performances of LSTM and GRU increased by 1.12% and 3%, respectively, compared to those when sentiment polarities were not used, and these differences were statistically significant. These results also confirmed the differences through a binomial test for the win/loss of the two model outcomes, and the results were compared using the periodical model comparison method utilized in previous studies. Despite these comparisons, it was shown that using sentiment polarities from social data for prediction is more significant. Additionally, these results indicate that it is possible to predict the number of confirmed cases by continuously monitoring both the number of confirmed cases and the sentiment state.

Through continuous monitoring of social sentiment states, it is possible to develop and adjust policies that reflect changes in public perception. Policymakers can evaluate the effectiveness of policies based on real-time sentiment data and swiftly adjust them as needed to meet public demands. In addition, it is possible to prevent the spread of misinformation and gain public trust. Based on the results of social media sentiment analysis, tailored messages can be crafted and distributed to the public, and communication strategies can be established to promptly counteract misinformation.

However, the study has limitations in terms of the data and models used therein. In the collection of social data, the data of other media and news cannot be included by analyzing only Twitter data. In case of the model, the comparative analysis results presented herein consider only the DNN, LSTM, and GRU ML models. In addition, as an opinion mining method, only sentiment analysis was used considering the appearance frequencies of positive/negative keywords in the sentiment dictionary.

In the future, studies should be to collect large volumes of high quality social data, conduct experiments using predictive models that are based on methods different from those used in this study, and present a model that predicts a week or longer ahead to produce practical results. In addition to sentiment analysis, the opinion methodology can be confirmed through future tasks to derive results by using various recently emerged models, including DL.

This study started with the aim of improving the prediction of the number of confirmed patients by incorporating sentiment polarities from social data. The results confirmed that including polarity allowed for statistically significantly higher accuracy in predictions compared to excluding polarity. While many previous studies relied solely on quantitative social data, this study highlighted the importance of qualitative opinions from social data in predicting the number of confirmed infectious disease patients. Therefore, it underscores the need for further research using social data and opinion mining in the field of infectious disease prediction.

## Supporting information

S1 Data Collected social data1. (XLSX)

S1 File Collected social data2 and Korea’s daily number of confirmed cases. (ZIP)
